# Discrimination of *Chrysanthemum* Varieties Using Hyperspectral Imaging Combined with a Deep Convolutional Neural Network

**DOI:** 10.3390/molecules23112831

**Published:** 2018-10-31

**Authors:** Na Wu, Chu Zhang, Xiulin Bai, Xiaoyue Du, Yong He

**Affiliations:** 1 College of Biosystems Engineering and Food Science, Zhejiang University, Hangzhou 310058, China; nawu018@zju.edu.cn (N.W.); chuzh@zju.edu.cn (C.Z.); xlbai@zju.edu.cn (X.B.); xydu@zju.edu.cn (X.D.); 2 Key Laboratory of Spectroscopy Sensing, Ministry of Agriculture and Rural Affairs, Zhejiang University, Hangzhou 310058, China; 3 State Key Laboratory of Modern Optical Instrumentation, Zhejiang University, Hangzhou 310058, China

**Keywords:** hyperspectral imaging, variety discrimination, *Chrysanthemum*, deep convolutional neural network

## Abstract

Rapid and accurate discrimination of *Chrysanthemum* varieties is very important for producers, consumers and market regulators. The feasibility of using hyperspectral imaging combined with deep convolutional neural network (DCNN) algorithm to identify *Chrysanthemum* varieties was studied in this paper. Hyperspectral images in the spectral range of 874–1734 nm were collected for 11,038 samples of seven varieties. Principal component analysis (PCA) was introduced for qualitative analysis. Score images of the first five PCs were used to explore the differences between different varieties. Second derivative (2nd derivative) method was employed to select optimal wavelengths. Support vector machine (SVM), logistic regression (LR), and DCNN were used to construct discriminant models using full wavelengths and optimal wavelengths. The results showed that all models based on full wavelengths achieved better performance than those based on optimal wavelengths. DCNN based on full wavelengths obtained the best results with an accuracy close to 100% on both training set and testing set. This optimal model was utilized to visualize the classification results. The overall results indicated that hyperspectral imaging combined with DCNN was a very powerful tool for rapid and accurate discrimination of *Chrysanthemum* varieties. The proposed method exhibited important potential for developing an online *Chrysanthemum* evaluation system.

## 1. Introduction

As one of the most popular flowers throughout the world, *Chrysanthemum* has a long planting history in China. The excellent ornamental, edible and medicinal values make *Chrysanthemum* used in many different forms. *Chrysanthemum* tea is one of the most commonly consumed teas for Chinese consumers. The chemical components such as flavonoids and polysaccharides rich in *Chrysanthemum* tea have antioxidant and antibacterial properties, which can relieve cell damage and improve body immunity [1,2]. The nutritional qualities of *Chrysanthemum* tea are affected by many factors, including climate, soil, water, cultivation management and post-harvest treatment, being the variety a determinant factor. Due to differences in content of chemical compositions, different varieties of *Chrysanthemum* tea have specific effects on human bodies. With the frequent mixing of *Chrysanthemum* from different varieties in the market in recent years, the purity of *Chrysanthemum* is difficult to guarantee. Thus, an appropriate method for discrimination of *Chrysanthemum* varieties is needed. The appearance characteristics such as color, flower diameter, petal shape often serve as the basis to identify *Chrysanthemum* varieties. This visual inspection method is subjective and requires professional knowledge. Some other approaches like high performance liquid chromatography (HPLC) combined with photodiode array detection, employed to determine the quality attributes, are destructive, time consuming, and can only handle very small number of samples [3]. Therefore, a rapid and accurate method would be advantageous when large number of *Chrysanthemum* samples need to be classified.

Near-infrared spectroscopy (NIRS), as a potential technology for rapid measurement, has been widely used in different fields such as geographical origin discrimination of agricultural products [4], quality assessment of agricultural seeds [5], variety identification of Chinese herbal medicines [6]. However, the samples needed to be shattered into powder when using this technology, making extraction of external space information difficult. Moreover, the sample size in these studies was very small which could not cover a broad variation. In contrast to NIRS, hyperspectral imaging (HSI) perfectly integrating visible/near-infrared spectroscopy and optical imaging in one system, can acquire both spectral information and spatial information. The capacity of collection spectra of multiple samples in one scan simultaneously gives HSI the property of batch detection, which makes the practical application possible. In addition, the spectra and the corresponding location of each pixel in image recorded by HSI can be employed to visualize the variety and chemical composition distribution of the samples.

To extract spectral and spatial information of a sample, hyperspectral image contains hundreds of contiguous wavebands for each pixel. Multivariate analysis methods, including spectral and image preprocessing, variable extraction and selection, model building and analysis, are often utilized to process this kind of data [7,8,9]. Currently, traditional machine learning methods combined with HSI have been widely used in variety identification of agricultural products [10,11,12,13,14], and multiple classification models were utilized, such as multiple logistic regression (MLR) [15], partial least squares discriminant analysis (PLS-DA) [16], support vector machine (SVM) [17], extreme learning machine (ELM) [18].

Deep learning, also known as representational learning, is a research focus in artificial intelligence nowadays. Among a variety of deep learning algorithms, deep convolution neural network (DCNN) aims to automatically extract abstract distributed features layer-by-layer. Various DCNNs has dramatically improved the state-of-the-art results in many vision tasks. In the field of hyperspectral image analysis, DCNN was first introduced in 2015 to classify hyperspectral sensing data [19]. In recent years, researchers have developed different DCNNs according to specific spectral analysis tasks, such as variety identification of rice seeds [20], disease detection of wheat *Fusarium* head blight [21], crop classification from remote sensing images [22]. It is of interest to further investigate if DCNN has the potential to discriminate the *Chrysanthemum* varieties.

The main objective of this study was to explore the feasibility of using HSI technique combined with DCNN for variety discrimination and visualization of *Chrysanthemum*. The specific objectives were to: (1) select important wavelengths that can contribute to identification of *Chrysanthemum* varieties, (2) develop appropriate DCNNS using full wavelengths and optimal wavelengths, (3) compare the results of DCNNs with traditional machine learning methods, including SVM and LR, (4) visualize the identification results of *Chrysanthemum* varieties using the optimal model.

## 2. Results

### 2.1. Overview of Spectra

**Figure 1:**
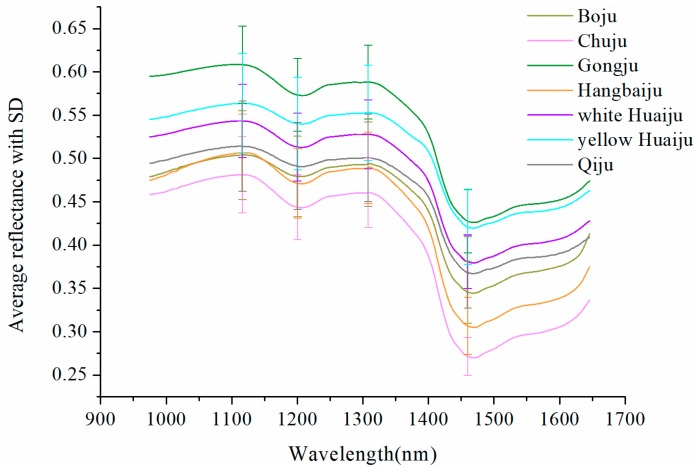
The average spectra of *Chrysanthemum* samples of seven varieties.

Figure 1 shows the mean spectra with standard deviation (SD) of *Chrysanthemum* samples of seven varieties. The shape of the reflectance curves was similar to that of *Chrysanthemum* in [23]. It can also be seen from the figure that the average spectra of seven *Chrysanthemum* varieties shared the consistent trend with similar peak and valley positions. However, slight differences could be observed from the average spectra of *Chrysanthemum* samples. The different chemical compositions and biochemical characteristics of these seven varieties resulted in these differences in spectral features. The peaks, around 1116 and 1308 nm, and valleys, around 1200 and 1460 nm, in spectral curves could be employed to discriminant the *Chrysanthemum* varieties. Among them, the two peaks and the valley at 1200 nm are attributed to the second overtone of C–H stretching [24,25], while the valley at 1460 nm (around 1450 nm) is attributed to the first overtone of O–H stretching [25]. In addition, it could be clearly observed that the spectral curves of Boju and Hangbaiju are very close and partially overlapping in the range of 975–1200 nm, indicating that the chemical compositions of these two varieties are similar.

### 2.2. Principal Component Analysis

**Figure 2:**
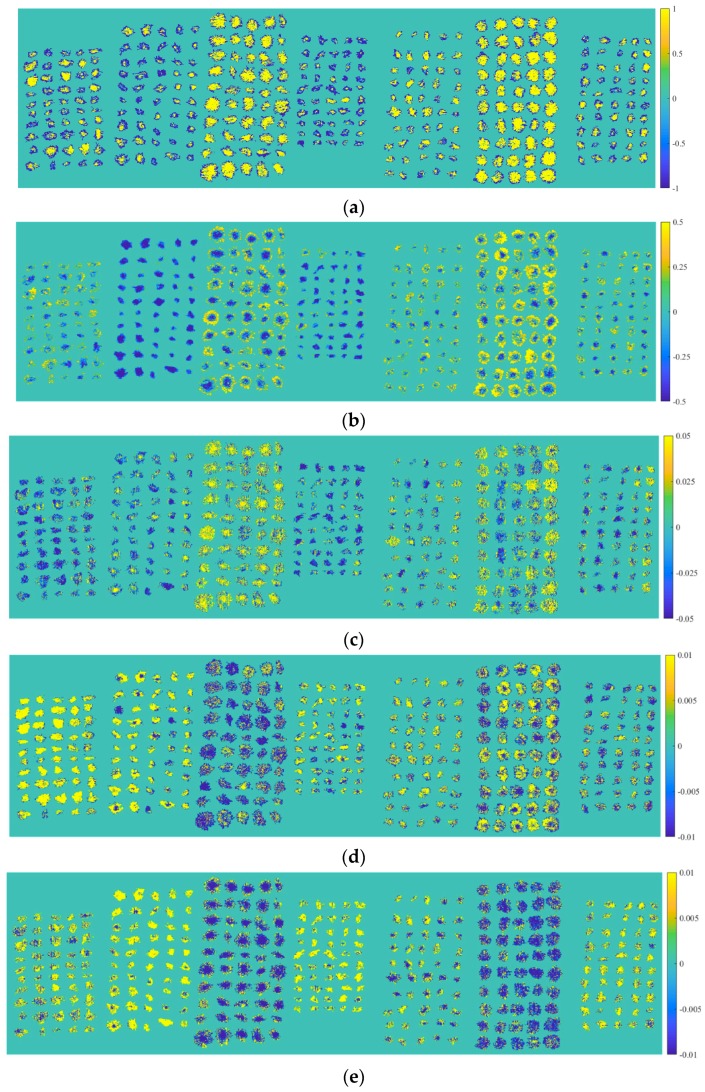
Score images of the first five PCs of seven *Chrysanthemum* varieties (from left to right: Boju, Chuju, Gongju, Hangbaiju, white Huaiju, yellow Huaiju, and Qiju): (**a**) PC1; (**b**) PC2; (**c**) PC3; (**d**) PC4; (**e**) PC5.

In the field of spectral analysis, PCA is often used as a method for qualitative analysis. In this study, PCA was employed to explore the differences between seven *Chrysanthemum* varieties. A hyperspectral image for each variety was random selected from the testing set for PCA. The first five PCs reflected 99.95% of information in original spectral data (96.61%, 3.17%, 0.11%, 0.04%, 0.02% for PC1, PC2, PC3, PC4, PC5, respectively). Thus, these five PCs of seven hyperspectral images were extracted. The pixels with PC value in sample region together with pixels with zero value in black background formed the final score images illustrated in Figure 2, from which the scores of *Chrysanthemum* samples of each variety were displayed intuitively, and some varieties could be preliminarily distinguished through combining these five PCs. For example, Boju could be highlighted because of the high scores of most sample pixels in PC4, which caused the samples to appear yellow. Due to the negative scores of most pixels in PC2, it was clear to discriminate Chuju and Hangbaiju from other *Chrysanthemum* varieties. However, the further discrimination between Chuju and Hangbaiju was difficult. In addition, it was easy to distinguish Gongju and yellow Huaiju from other *Chrysanthemum* varieties in PC1 and PC5, since most pixels of these two varieties had high scores in PC1 and negative scores in PC5. And Gongju could be further identified in PC4 for its negative scores. White Huaiju and Qiju having the same clustering pattern as some other varieties could not be identified. To distinguish all *Chrysanthemum* varieties, discriminant models need to be built for quantitative analysis in further study.

### 2.3. Selection of Optimal Wavelengths

**Figure 3:**
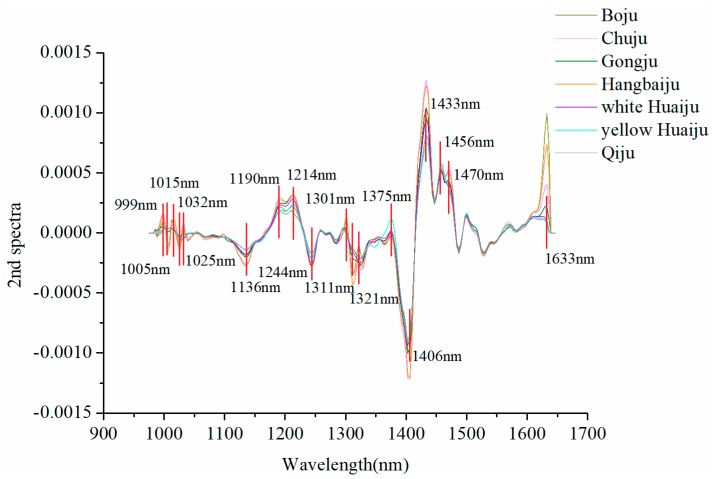
The 2nd derivative spectra and the selected optimal wavelengths.

In order to remove the redundancy information contained in hyperspectral images and improve the classification performance of *Chrysanthemum* varieties, second derivative (2nd derivative) method was introduced to select optimal wavelengths from full wavelengths. Figure 3 shows the 2nd derivative spectra of average spectra of seven varieties. There are multiple high peaks and low valleys in the 2nd derivative spectra, and the wavelengths with large differences between *Chrysanthemum* varieties were selected as optimal wavelengths for discrimination. Finally, eighteen optimal wavelengths were selected in total. Among them, the absorption bands at approximately 999, 1005, 1015, 1025 and 1032 nm are attributed to the second overtone of N−H stretching [17]. The wavelengths between 1136 nm and 1311 nm (1136, 1190, 1214, 1244, 1301, 1311 nm) are related to the second overtone of C-H stretching [17,26]. The selected wavelengths of 1321 and 1375 nm are associated with the first overtone of C–H combination bands [27]. The bands at 1406, 1433 and 1456 nm present the first overtone of O–H stretching [27]. The bands at 1470 nm (around 1480 nm) is ascribed to the second overtone of O-H stretching [25,28]. The peak at 1633 nm (around 1630 nm) is attributed to the aromatic C-H bands [29]. These wavelengths carrying the category information are closely related to the constituent differences of chemical composition of different *Chrysanthemum* varieties.

### 2.4. Discrimination Results of Different Models

Discriminant models using full and optimal wavelengths were built by SVM, LR, and DCNN for quantitative analysis. The classification accuracies of different models and corresponding parameters were summarized in Table 1. As can be seen in Table 1, SVM, LR and DCNN models all achieved good classification results on both training set and testing set. For full wavelengths, the accuracies of these three models on the training set were greater than 99%, and the accuracies on the testing set were more than 94%. The classification capacity of DCNN was better than those of SVM and LR, showing accuracies of close to 100% on both training set and testing set. Being able to learn deep spectral features automatically, DCNN could provide excellent classification performance.

Since a large amount of redundant information existed in full wavelengths, the optimal wavelengths were often extracted in previous spectral analysis to improve the robustness of the model. In this study, 2nd derivative method was introduced to select the optimal wavelengths. The accuracies of the three models based on optimal wavelengths were slightly lower than those based on full wavelengths. Consistent with the results based on full wavelengths, the best results were still obtained by DCNN model with an accuracy of 98.45% on training set and 94.27% on testing set. LR was most sensitive to wavelength reduction that led to the largest drop of accuracy on testing set. Due to the removal of a part of spectral information, a slight accuracy reduction was understandable. However, the fact that SVM and LR based on optimal wavelengths achieved lower accuracies than DCNN based on full wavelengths further proved that the deep spectral features learnt by DCNN were more distinguishable than the selected feature wavelengths.

In summary, DCNN achieved better classification performance than the traditional machine learning algorithms, including SVM and LR. The overall results indicated that hyperspectral imaging combined with DCNN was feasible to distinguish *Chrysanthemum* varieties. Without any optimal wavelengths extraction, DCNN based on full wavelengths is a very reliable model and is available for identification of more *Chrysanthemum* varieties in future.

### 2.5. Visualization of Chrysanthemum Variety Classification

**Figure 4:**
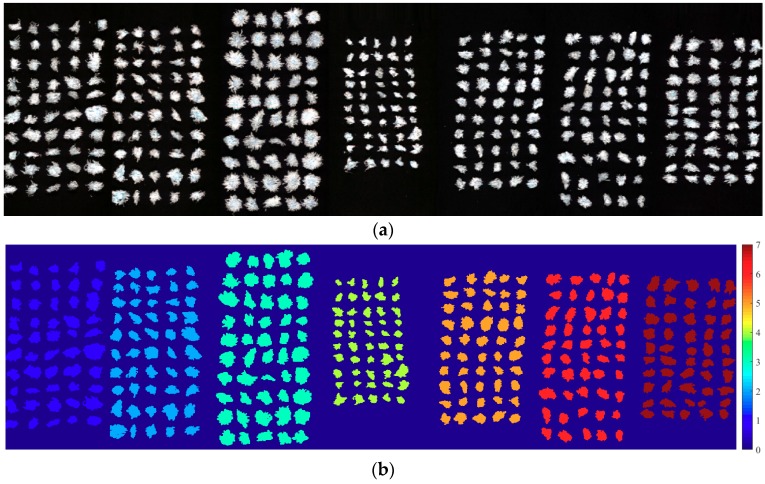
Visualization of *Chrysanthemum* varieties (from left to right: Boju, Chuju, Gongju, Hangbaiju, white Huaiju, yellow Huaiju, and Qiju): (**a**) Original grayscale images; (**b**) The classification maps.

In order to discriminant the *Chrysanthemum* varieties more intuitively, the optimal model, DCNN based on full wavelengths, was used to visualize the classification of *Chrysanthemum* varieties in this study. A hyperspectral image for each variety was randomly selected from testing set. The original grayscale images of seven varieties are shown in Figure 4a. Although some *Chrysanthemum* varieties differed in size from others, it was difficult to identify all varieties according to the external phenotype. The corresponding classification maps were displayed in Figure 4b. The low resolution of hyperspectral images and the application of some morphological operations during image segmentation resulted in some changes of *Chrysanthemums*’ shape. However, the main patterns and positions of the *Chrysanthemums* were clearly expressed on the classification maps. It was easy to distinguish different *Chrysanthemum* varieties according to the colors. For these randomly selected hyperspectral images, DCNN based on full wavelengths classified all samples correctly. That is to say, DCNN achieved an accuracy of 100%, which is consistent with the quantitative analysis. The visualization results indicated that hyperspectral imaging combined with DCNN provided a rapid, accurate and intuitive way to distinguish *Chrysanthemum* varieties, which is a potential tool for identifying and locating more *Chrysanthemum* varieties.

## 3. Discussion

Influenced by growth environment, cultivation management, picking period and other factors, the chemical compositions of *Chrysanthemums* from same variety may vary greatly. For example, there are significant differences in total polysaccharide content and total flavonoid content between *Chrysanthemum* picked in different periods. As a result of these differences, their pharmacological properties and prices vary widely [23,30]. To include these variations, large-scale samples need to be collected. In previous studies, the classification of *Chrysanthemum* varieties has been reported. A total of 200 samples including five cultivars of *Chrysanthemum* were classified using a multispectral imaging system in [31]. To identify three kinds of white *Chrysanthemum*, a near infrared spectroscopy system was employed to collect the spectra of 139 samples and 92 spectra were selected as calibration set to build the identification model in [32]. In this study, a total of 11,038 samples of seven *Chrysanthemum* varieties were classified using hyperspectral imaging technology. The characteristic of batch detection of hyperspectral imaging makes it possible to acquire large-scale samples, which also provides favorable conditions for the application of deep learning.

As a research focus in machine learning, deep learning has been gradually applied in the field of spectral analysis. DCNN is a typical deep learning algorithm that learns abstract features through multiple convolutional layers. The large-scale samples obtained by hyperspectral imaging technology enable DCNN to fully exploit its advantages and automatically learn the deep spectral features contained in hyperspectral images. In previous studies on spectral analysis, the optimal wavelengths were commonly selected manually and then modeled using traditional machine learning algorithms such as SVM, LR, and KNN [16]. However, deep learning algorithms often achieved good classification results without additional feature selection [33,34]. In this study, DCNN and two traditional machine learning algorithms using full wavelengths and optimal wavelengths were compared. The results showed that DCNN based on full wavelengths achieved the best performance. This further illustrated that DCNN can discriminate *Chrysanthemum* varieties more accurately since it can learn deep spectral features through multiple hidden layers automatically. More *Chrysanthemum* varieties need to be collected to develop a *Chrysanthemum* variety identification instrument. In addition, in order to further evaluate the quality of *Chrysanthemum*, a comprehensive research need to be conducted in future. Combining the advantages of hyperspectral imaging and DCNN, an on-line detection system of *Chrysanthemum* varieties and quality could be developed.

## 4. Materials and Methods

### 4.1. Sample Preparation

Seven varieties of dried *Chrysanthemum*, including Boju, Chuju, Gongju, Hangbaiju, white Huaiju, yellow Huaiju, and Qiju, were collected for our experiment. Among them, Boju, Chuju, and Gongju were bought from the local tea sales companies in Bozhou, Chuzhou and Huangshan, Anhui Province, China, respectively. Hangbaiju were bought from the local market in Hangzhou, Zhejiang Province, China. The two varieties of Huaiju and Qiju were bought from the local tea sales companies in Jiaozuo, Henan Province and Anguo, Hebei Province, China, respectively. All *Chrysanthemums* were harvested in 2017 and had a similar dry state.

In total, 1600, 1500, 1643, 1600, 1500, 1590, 1605 samples were obtained for Boju, Chuju, Gongju, Hangbaiju, white Huaiju, yellow Huaiju, and Qiju, respectively. The dataset of each variety was randomly divided into a training set and a testing set at a ratio of 3:1. Therefore, there were 8280 samples in the training set and 2758 samples in the testing set. All *Chrysanthemum* samples were assigned a category label. Boju, Chuju, Gongju, Hangbaiju, white Huaiju, yellow Huaiju, and Qiju were assigned from 1 to 7, respectively.

### 4.2. Hyperspectral Image Acquisition and Correction

Hyperspectral images of *Chrysanthemums* were acquired using a near-infrared HSI system. This system consists of a group of devices interacting to each other: an imaging spectrograph (ImSpector N17E; Spectral Imaging Ltd., Oulu, Finland) with a spectral range of 874–1734 nm, a high-performance CCD camera assembled with a camera lens (OLES22; Specim, Spectral Imaging Ltd., Oulu, Finland) having a resolution of 326 × 256 (spatial × spectral) pixels, two 150-W tungsten halogen lamps (3900e Lightsource; Illumination Technologies Inc.; West Elbridge, NY, USA) regarded as the illumination unit, and a conveyer belt controlled by a stepped motor (Isuzu Optics Corp., Zhubei, Taiwan) used for moving samples.

To obtain non-deformable and clear hyperspectral images, dried *Chrysanthemums* were placed on the conveyer belt, and the distance between the camera lens and the conveyer belt, the exposure time of the camera, and the speed of the conveyer belt along X-axis were adjusted to 25 cm, 4 ms and 19.5 mm/s, respectively. The acquired hyperspectral images of *Chrysanthemums* were composed of 256 spectral channels with a spectral resolution of 5 nm.

To reduce the effects of dark current and obtain the reflectivity of samples, raw hyperspectral images *I_raw_* should be corrected with the white reference image and black reference image using the following Equation (1):

(1) $$I_{c}=\frac{I_{raw}-I_{dark}}{I_{white}-I_{dark}}\;$$

where *I_c_* is the hyperspectral image after corrected, *I_white_* is the hyperspectral image of a white Teflon tile with nearly 100% reflectance, *I_dark_* is acquired by covering the camera lens with its opaque cap. *I_raw_*, *I_white_*, *I_dark_* are obtained under the same condition during samples collection.

### 4.3. Spectra Extraction and Pretreatment

Before spectra extraction, the region of interest (ROI), each *Chrysanthemum* sample region, need to be segmented from the black background. A threshold segmentation procedure was conducted on the gray image at 1119 nm where the contrast between the sample regions and the background reached the maximum value, and then the obtained binary mask was applied on the gray images at other wavelengths. After getting ROI of each *Chsrysanthemum* sample, the spectrum of each pixel in each ROI with a spectral range of 874–1734 nm was extracted. Due to the instability of hyperspectral imaging system at the start and end of sample collection, the beginning and the end of the spectral data contained random noise. Thus, the middle 200 wavelengths from 975 nm to 1646 nm were used for analysis. To further reduce the spectral noise and improve the signal-to-noise ratio, wavelet transform (WT) with decomposition scale of 3 and basis function of Daubechies 6 was employed to smooth the pixel-wise spectra. Finally, the preprocessed pixel-wise spectra in each ROI were averaged and the mean spectrum of each *Chrysanthemum* sample was used for discrimination analysis.

### 4.4. Chemometrics Analysis

PCA is a powerful tool to reduce the dimensionality of high-dimensional data. More importantly, PCA can remove noise and discover patterns inherent data through dimensionality reduction. In spectral analysis, each specific wavelength regarded as a feature variable forms the spectral matrix. PCA is applied to this matrix, and projected the original spectral variables into a new coordinate system by maximizing the sample variance. The variables in the new coordinate system called PCs are a linear transformation of original spectral variables and are orthogonal to each other. The PCs are arranged in descending order of interpreted variance and the first few PCs can reflect most of variance inherent in original matrix. From the score images of PCs, it is possible to identify the pattern difference between different categories of data.

Collinearity and redundancy exist among the contiguous wavelengths in hyperspectral image. Optimal wavelength selection is an efficient way to extract wavelengths that are beneficial for classification. 2nd derivative is a widely-used wavelength selection method, which can highlight spectral change [35]. Subtle changes in original spectra can be projected into the peaks and the valleys in 2nd derivative spectra. The wavelengths corresponding to the peaks and valleys with large difference between spectra could be selected as the optimal wavelengths to discriminant different sample categories.

### 4.5. Discriminant Methods

To classify the *Chrysanthemum* samples correctly, a DCNN was built as the discriminant model. Traditional machine learning methods, including SVM and LR, were introduced as contrast methods.

#### 4.5.1. Support Vector Machine

SVM is a supervised machine learning approach, widely used in spectral data classification. The basic principle of SVM is to find the optimal hyperplane that maximizes the interval between the positive and negative samples in training set. To solve the nonlinear problem, kernel function is introduced into SVM. The hidden mapping of samples from original feature space into a new high-dimension space using kernel function can make the samples change from the linear indivisible state to a linear separable state [36]. Among the kernel functions, radial basis function (RBF) is efficient to deal with nonlinear classification problem. In this study, RBF was selected as the kernel function of SVM. To obtain a satisfactory classification performance, penalty coefficient *c* and the kernel parameter *g* could be determined using a simple grid-search procedure.

#### 4.5.2. Logistic Regression

LR is a commonly-used pattern recognition approach to solve classification problem using regression-like method. Sigmoid function is utilized to map the real value predicted by linear regression model into the value in range 0–1. The output of sigmoid function is treated as the predicted category probability. When solving binary classification problem (labeled by 0 and 1), the sample with a value greater than or equal to 0.5 is classified as category 1, otherwise assigned to category 0. When solving multi-classification problem, multiple one-to-many binary classification models are combined. Structural risk loss is employed as the objective function to be optimized [15]. The penalty item *pi* can be set to L1 regularization or L2 regularization to reduce the overfitting risk. The inverse of regularization coefficient *c’* can be adjusted, while small *c’* causes strong regularization. The optimization algorithms *optimize_algo*, including newton-cg, lbfgs, liblinear, sag, can be selected to optimize the loss function according to the classification performance.

#### 4.5.3. Deep Convolutional Neural Network

A DCNN was further developed to discriminate the *Chrysanthemum* varieties, and its performance was compared with that of SVM and LR. A typical DCNN consists of convolutional layers to extract the local features, pooling layers to reduce the size of parameters and fully-connected layers to output the classification results.

**Figure 5:**
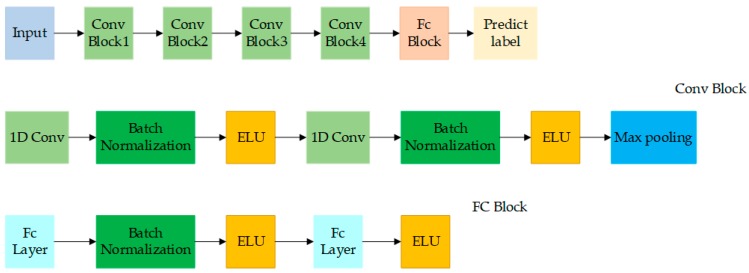
The structure of DCNN based on full wavelengths.

The structure of our designed DCNN for full wavelengths shown in Figure 5 contained four convolutional modules and two full connected layers. Each convolutional module included two convolutional layers followed by a max pooling layer. The number of filters in the first convolutional module was set to 32, and was doubled as the modules going deeper. To process one-dimensional spectral data, the commonly-used two-dimensional convolution kernels were replaced by one-dimensional convolution kernels. The trick of using two consecutive 1 × 3 kernels instead of a 1 × 5 kernel was inspired by VGGNet to decrease the number of parameters while increasing the network depth [34]. Each convolution kernel was acted on the local region of the feature maps of the upper layer, and all regions were processed by the same kernel. This mechanism allowed DCNN to quickly learn the local spectral features in parallel. The max pooling layer with a kernel of 1 × 2 was used to reduce the number of feature maps to the half. The stride and padding of all the filters were set to 1. The two full connected layers were used to combine the features output by the last convolution module.

Exponential linear unit (ELU) was selected as the activation function in this study due to its better performance than rectified linear unit (RELU), which was consistent with the results in [20,37]. The right linear part allows ELU to mitigate the gradient disappearance like other activation functions. The left soft-saturated part allows ELU to push the mean of the active unit closer to 0, thereby reducing the offset effect and making ELU more robust to input variations and noise. Combining the advantages of these two parts, ELU can speed up the training process and improve the classification accuracy. The expression of ELU is as shown in Equation (2):

(2) $$f(x)=\{\begin{array}{cc} x & x\geq 0\\ \alpha (\exp(x)-1) & x<0 \end{array}\;$$

As an important achievement of deep learning in recent years, Batch Normalization has been widely proved to be effective and important [38]. For each neuron in hidden layers, Batch Normalization forces the input distribution closing to the saturation region back to the standard normal distribution to reduce the offset effect like ELU. The consistent scale of data in each layer and each dimension makes parameter adjustment efficient. This accelerates the convergence process, reduces the possibility of overfitting and improves the classification performance. In this study, Batch Normalization was inserted before each ELU (except the last fully connected layer).

At the end of DCNN, a softmax function was introduced to transform the output of last fully-connected layer to the value in range 0–1, which represents the relative probability between different categories. Then, the cross-entropy loss was chosen as the objective function to evaluate the difference between the output of DCNN and the ground-truth in training phase. The cross-entropy loss function can be defined by Equation (3):

(3) $$Loss=-\sum_{x}p(x)\log q(x)\;$$

where x is the input of DCNN, p(x) is the probability value of expected output, q(x) is the probability value predicted by DCNN. A Stochastic Gradient Descent (SGD) optimizer with a learning rate of 0.001 and a momentum of 0.9, was used to minimize the cross-entropy loss function during training. And the batch size was set to 256. The network structure for optimal wavelengths was similar. The number of convolution modules *num_convs*, the number of convolution kernels in the first convolution module *num_first_kernels*, and the iterations of network training *epoch* should be adjusted according to the classification performance.

### 4.6. Chrysanthemum Varieties Visualization

Visualization of *Chrysanthemum* varieties facilitates intuitive and rapid inspection of *Chrysanthemum* varieties by industrial producers and market regulators. The advantage of hyperspectral imaging to obtain spatial and spectral information simultaneously makes visualization of *Chrysanthemum* varieties possible. To build the classification maps, the average spectrum of each sample in hyperspectral image was input into the classification model, and the obtained label was mapped back to each pixel of the corresponding sample in hyperspectral image. In this study, the optimal discriminant model based on hyperspectral imaging was selected to visualize the spatial distribution of *Chrysanthemum* varieties. Different *Chrysanthemum* varieties were assigned to different colors on the chemical imaging maps, which is beneficial for identifying the specific *Chrysanthemums* whose varieties are different from that of most *Chrysanthemums*.

### 4.7. Software

ENVI 4.6 (ITT Visual Information Solutions, Boulder, CO, USA) was used to crop the *Chrysanthemum* samples from the irrelevant background in hyperspectral images. MATLAB R2018a (The MathWorks, Natick, MA, USA) was used to extract and preprocess the spectral data from hyperspectral images. PCA for pattern recognition between different varieties was also implemented with MATLAB R2018a. Unscrambler 10.1 (CAMO AS, Oslo, Norway) was used to extract the optimal wavelengths by 2nd derivative method. Discriminant models including SVM, LR and DCNN were implemented using python language with Spyder3.2.6 (Anaconda, Austin, TX, USA). The famous machine learning library sklearn (http://scikit-learn.org/stable/) and convenient deep learning framework Pytorch (Facebook, Menlo Park, CA, USA) were used during programming. All software tools were carried out on the software platform of win10 64-bit operating system and the hardware platform of a computer with Inter(R) Core (TM) i5-8500 3.00 HZ CPU and 8 G memory.

## 5. Conclusions

Hyperspectral imaging combined with DCNN was used to distinguish *Chrysanthemum* varieties. The qualitative analysis of PCA showed that different *Chrysanthemum* varieties could be preliminarily distinguished according to the score images. The optimal wavelengths with certain distinguishing ability were selected by 2nd derivative method. The performance of SVM, LR, and DCNN models using full wavelengths and optimal wavelengths were compared, and the performance of models based on full wavelengths were superior to those based on optimal wavelengths. DCNN based on full wavelengths obtained the best classification results, indicating that the deep spectral features automatically learned by DCNN were more beneficial for discrimination than the artificially selected optimal wavelengths. The classification maps of *Chrysanthemum* varieties formed by DCNN made the spatial distribution of *Chrysanthemum* varieties to be displayed in an intuitive manner, showing great potential of rapid detection of large-scale samples in industrial production. The overall results indicated that the characteristics of non-destructive and batch detection of hyperspectral imaging and the ability of automatically learning deep features of DCNN were the key factors for rapid and accurate discrimination of *Chrysanthemum* varieties. This study provides a new idea for identification of *Chrysanthemum* varieties.

## Figures and Tables

**Table 1:** Discrimination results of *Chrysanthemum* varieties by different models using full wavelengths and optimal wavelengths.

Models	Full Wavelengths			Optimal Wavelengths		
	Parameters ^1^	Training	Testing	Parameters	Training	Testing
SVM	(10^6^, 10^−5^)	99.83%	94.02%	(10^7^, 10^−4^)	98.26%	90.03%
LR	(L2, 100, liblinear)	99.34%	96.59%	(L2, 100, liblinear)	94.35%	85.75%
DCNN	(4, 32, 93)	99.98%	99.98%	(3, 32, 125)	98.45%	94.27%

^1^ The parameters of the discriminant models. (*c*, *g*) for SVM, (*pi*, *c’*, *optimize_algo*) for LR, and (*num_convs*, *num_first_kernels*, *epoch*) for DCNN.
